# Infectivity and structure of SARS-CoV-2 after hydrogen peroxide treatment

**DOI:** 10.1128/mbio.03994-24

**Published:** 2025-04-21

**Authors:** Saba R. Aliyari, Guodong Xie, Xian Xia, Lulan Wang, Z. Hong Zhou, Genhong Cheng

**Affiliations:** 1 Department of Microbiology, Immunology and Molecular Genetics, University of California, Los Angeles (UCLA), Los Angeles, California, USA; 2 California NanoSystems Institute, UCLA, Los Angeles, California, USA; Griffith University-Gold Coast Campus, Gold Coast, Queensland, Australia

**Keywords:** hydrogen peroxide, SARS-CoV-2, spike proteins, cryo-ET

## Abstract

Hydrogen peroxide (H_2_O_2_) exhibits broad-spectrum antiviral activity and is commonly used as an over-the-counter disinfecting agent. However, its potential activities against SARS-CoV-2 have not been systematically evaluated, and mechanisms of action are not well understood. In this study, we investigate H_2_O_2_’s antiviral activity against SARS-CoV-2 infection and its impact on the virion’s structural integrity as compared to the commonly used fixative agent paraformaldehyde (PFA). We show that H_2_O_2_ rapidly and directly inactivates SARS-CoV-2 with a half-maximal inhibitory concentration (IC50) of 0.0015%. Cryogenic electron tomography (cryo-ET) with subtomogram averaging reveals that treatment with PFA induced the viral trimeric spike protein (S) to adopt a post-fusion conformation, and treatment of viral particles with H_2_O_2_ locked S in its pre-fusion conformation. Therefore, H_2_O_2_ treatment likely has induced modifications, such as oxidation of cysteine residues within the S subunits of the spike trimer that locked them in their pre-fusion conformation. Locking of the meta-stable pre-fusion trimer prevents its transition to the post-fusion conformation, a process essential for viral fusion with host cells and entry into host cells. Together, our cellular, biochemical, and structural studies established that hydrogen peroxide can inactivate SARS-CoV-2 in tissue culture and uncovered its underlying molecular mechanism.

**IMPORTANCE:**

Hydrogen peroxide (H_2_O_2_) is the commonly used, over-the-counter antiseptic solution available in pharmacies, but its effect against the SARS-CoV-2 virus has not been evaluated systematically. In this study, we show that H_2_O_2_ inactivates the SARS-CoV-2 infectivity and establish the effective concentration of this activity. Cryogenic electron tomography and sub-tomogram averaging reveal a detailed structural understanding of how H_2_O_2_ affects the SARS-CoV-2 spike in comparison with that of the commonly used fixative PFA under identical conditions. We found that PFA promoted a post-fusion conformation of the viral spike protein, while H_2_O_2_ could potentially lock the spike in its pre-fusion state. Our findings not only substantiate the disinfectant efficacy of H_2_O_2_ as a potent agent against SARS-CoV-2 but also lay the groundwork for future investigations into targeted antiviral therapies that may leverage the virus’ structural susceptibilities. In addition, this study may have significant implications for developing new antiviral strategies and improving existing disinfection protocols.

## INTRODUCTION

The first two decades of the new millennium have witnessed unprecedented global challenges posed by viral infections, with a series of novel and re-emerging pathogens, including severe-acute-respiratory-syndrome coronavirus (SARS-CoV), Middle East respiratory syndrome (MERS), Zika virus (ZIKV), and SARS-CoV-2, the virus responsible for COVID-19. Most notably, emerging in less than 20 years after SARS-CoV, SARS-CoV-2 is a novel coronavirus believed to have originated in bats and subsequently transmitted to humans, likely through an intermediate animal host. The impact of SARS-CoV-2 introduction has been profound, with millions of lives lost (more than 7 million as of September 2024) (1). One thing certain to occur both from public health history and epidemiology research is a new SARS-CoV-like pandemic in the future. As such, efforts to find a cheap, readily available, and wide-spectrum antiviral treatment regimen in preparation for such future pandemics are a public health priority.

SARS-CoV-2 is a single-stranded, positive-sense RNA virus belonging to the *Coronaviridae* family. Its genome is approximately 30 kilobases in length and contains several ORFs that encode various viral structural and non-structural proteins. Four main structural proteins are Spike (S), Envelope (E), Membrane (M), and Nucleocapsid (N). These proteins play essential roles in viral entry, assembly, and immune evasion (1–5). Among them, the 600 kDa trimeric Spike (S) protein plays a pivotal role in establishing infection by SARS-CoV-2. The S protein exists as a trimeric glycoprotein protruding from the viral envelope. The trimeric nature of the S protein enhances its stability and avidity for receptor binding, contributing to the efficient infection of target cells. The S1 subunit of the S protein contains the receptor-binding domain (RBD), a crucial region essential for recognizing and binding to specific cell host receptors, primarily the angiotensin-converting enzyme 2 (ACE2). Cysteine residues are indispensable for the proper function of the SARS-CoV-2 spike protein, stabilizing its pre-fusion structure and enabling the structural transitions required for viral entry. RBD contains a total of nine cysteine residues, eight of which form four pairs of disulfide bonds. Three pairs (Cys336–Cys361, Cys379–Cys432, and Cys391–Cys525) in the core help with stabilizing the β sheet structure, and the Cys480–Cys488 pair connects the loops in the distal end of the RBM (6). The RBD is typically in a “closed” or “down” conformation, masking the binding site from host cell receptors. This metastable conformation prepares the virus for receptor engagement. Upon binding to the ACE2 receptor, the S1 subunit undergoes significant structural rearrangements and conformational changes. The receptor-binding sites (RBS) of the S1 are exposed only when the RBDs adopt an “up” conformation. In this conformation, the RBDs are in an open or exposed state, allowing them to bind to the host cell receptor. Following binding to the receptor, S2 undergoes further structural rearrangements. These include shedding of the S1 subunit and exposing the fusion peptide (FP). The FP then inserts into the target cell membrane, facilitating virus-host cell membrane fusion. Following fusion, the S protein adopts a needle-shaped post-fusion conformation characterized by three helices entwining coaxially. This structural transformation allows for the release of the viral genome into the host cell, initiating viral replication (6–9).

As we continue to navigate through the SARS-CoV-2-caused health crises, the search for effective strategies to combat viral spread has become paramount. One such strategy that has garnered attention is the use of hydrogen peroxide (H_2_O_2_) as a potential treatment against a wide array of viruses including SARS-CoV-2 (10–12). In the context of viral infections, H_2_O_2_ is considered a potent virucidal agent. The mechanism responsible for the virucidal action of H_2_O_2_ involves the production of reactive oxygen species (ROS) upon decomposition, which exerts oxidative stress on viral proteins, nucleic acids, and lipid membranes. This oxidative damage disrupts the structural integrity of the virus, rendering it incapable of infecting host cells (13, 14).

Three-dimensional (3D) structures from cryogenic electron microscopy (cryo-EM) and tomography (cryo-ET) have provided insights into the topography and structural changes in SARS-CoV-2 spikes and revealed the structural rearrangements of S protein during the viral entry process. It is well established that the methods used for viral sample preparation, as well as virus inactivation and fixation, can influence the distribution of conformational states of the spike protein, including the ratio between pre-fusion and post-fusion forms (15). For example, treatment with PFA generally fixes viral particles predominantly in the pre-fusion state (16–18). However, SARS-CoV-2 infection of cells overexpressing ACE2 has been shown to promote a shift toward the post-fusion state of the S protein upon fixation by PFA. When SARS-CoV-2 particles are inactivated with PFA in A549 cells overexpressing ACE2, the S protein exclusively adopts the post-fusion conformation (19). In addition, in Vero cells treated with PFA, both pre-fusion and post-fusion states of the spike protein have been observed (20). On the other hand, treatment with β-propiolactone (PBL) tends to lock the majority of S proteins in the post-fusion state (21). Interestingly, another study found that PBL inactivation of SARS-CoV-2 resulted in nearly equal distribution (42% pre-fusion, 48% post-fusion) of spike protein conformations (22). In this study, we set out to investigate the antiviral effects of H_2_O_2_ on SARS-CoV-2 and determine how H_2_O_2_ affects the structural rearrangements of the S protein. We compared H_2_O_2_ effects with those of PFA using cryo-EM and cryo-ET under identical conditions. Our results demonstrated that, when viral particles were treated with PFA, the S protein on the virion envelope existed in the post-fusion state. By contrast, when viral particles were fixed with H_2_O_2_, the S protein was found to be locked in the pre-fusion state. This observation suggested that the fixation method could potentially impact the conformational dynamics of the S protein. H_2_O_2_ likely induced modifications, particularly oxidation of cysteine residues within the RBD of the S protein. This oxidative modification may have resulted in the stabilization of the pre-fusion conformation, preventing the transition of the S protein to the post-fusion state essential for viral fusion and entry into host cells.

## MATERIALS AND METHODS

All the SARS-CoV-2-based experiments were performed at the UCLA BSL3 facility.

### Viruses

The recombinant SARS-CoV-2 (icSARS-CoV-2-mNG) expressing mNeonGreen (23) was a kind gift from the World Reference Center for Emerging Viruses and Arboviruses (WRCEVA), Department of Microbiology and Immunology, University of Texas Medical Branch through an MTA. SARS-CoV-2 variant of concerns SARS-CoV-2-α (SARS-CoV-2, Isolate hCoV-19/USA/OR-OHSU-PHL00037/2021-B.1.1.7), SARS-CoV-2-β (SARS-CoV-2, Isolate hCoV-19/USA/MD-HP01542/2021B.1.351), SARS-CoV-2-γ (SARS-CoV-2, Isolate hCoV-19/Japan/TY7-503/2021 P.1 or 20J/501Y.V3), and SARS-CoV-2-δ (SARS-CoV-2, Isolate hCoV-19/USA/PHC658/2021-B.1.617.2) were provided by Dr. Vaithilingarajai Arumugaswami, who obtained them from the BEI resources.

### SARS-CoV-2 viral RNA copy number assay

Supernatant (200 µL) was harvested from cells infected with SARS-CoV-2 and mixed with an equal volume of lysis buffer provided in the high pure viral RNA kit (Invitrogen) and RNA was extracted according to the kit’s instructions. The eluted RNA was subjected to RT-qPCR using the One Step TB Green PrimeScript RT-qPCR Kit II (Takara) and specific primers targeting the SARS-CoV-2 nucleocapsid protein (NP): Forward, 5′-TAATCAGACAAGGAACTGATTA-3′, and Reverse, 5′-CGAAGGTGTGACTTCCATG-3′.

RT-qPCR cycling conditions were 42°C for 5 min, 95°C for 10 s, and 40 cycles of 95°C for 5 s, followed by 60°C for 30 s.

### RT-qPCR

Cells were collected in Trizol, and RNA was isolated by standard isopropanol precipitation. RNA was quantified, and 1 µg of RNA was reverse transcribed using iScript (BioRad) according to the manufacturer’s instructions with random hexamer as primers. RT-qPCR analysis was done using the iCycler thermocycler using gene-specific primers (Bio-Rad). RT-qPCR was conducted in a final volume of 20 µL. Amplification conditions were as follows: 95°C (3 min), 40 cycles of 95°C (20 s), 55°C (30 s), and 72°C (20 s). Expression values were normalized to L32 (RPL32), and fold induction was normalized to untreated control. For detection of SARS-CoV-2 genomic RNA, the below primers targeting SARS-CoV-2 nucleocapsid protein (NP) were used: NP-Forward, 5′-TAATCAGACAAGGAACTGATTA-3′, and NP-Reverse, 5′-CGAAGGTGTGACTTCCATG-3′.

### Identification of CC_50_ and IC_50_ values

Twenty hours prior to the cytotoxicity assay, 8 × 10^3^ HeLa-ACE-2 cells were seeded in 96-Well White/Clear Bottom Plate, TC Surface (Thermo Fisher). Cells were treated with twofold serial dilutions of H_2_O_2_ for 1 hour with or without catalase for 5 minutes, and cells were washed with 1× PBS, followed by the addition of fresh media. The cell viability was determined using the Cell Titer-Glo luminescent cell viability assay (Promega). IC_50_ and CC_50_ values were calculated by non-linear regression analysis using GraphPad 5.

### SARS-CoV-2 production, purification methods for structural studies

To expand and purify SARS-CoV-2 particles, we adapted protocols from Yao et al. (18) and Ke et al. (17) with some modifications. In brief, Vero-E6 cells were cultured to 80% confluency and then infected with SARS-CoV-2 at a MOI of 0.01 and incubated at 37°C in a 5% CO₂ incubator. At 60 hours post-infection (HPI), the medium from the infected cells was collected and subjected to three freeze-thaw cycles, followed by centrifugation at 10,000× *g* for 10 minutes at 4°C to pellet and remove cell debris. The resultant sample was taken for plaque assay to quantify the viral titer. Half of the pre-cleared media containing the virus was inactivated using 4% PFA, while the other half was treated with 3% H_2_O_2_. Fixation was conducted at room temperature for 3 hours, followed by overnight incubation at 4°C.

Non-infectivity of these treated virion samples was verified by the following steps: an aliquot of the fixed particles was used to infect fresh cells. At 24 HPI, the supernatant from these infected cells was collected for plaque assay, and RNA was extracted from the cell lysate for subsequent RT-qPCR analysis.

For cryo-EM studies, the above virion samples were layered onto a 20% sucrose cushion in 10 mM HEPES (pH 7.3) with 150 mM NaCl (HN buffer) and subjected to ultracentrifugation using a Beckman SW41Ti rotor at 100,000 × *g* for 2 hours at 4°C. Subsequently, the pellet was rinsed with HN buffer to eliminate any residual sucrose and then resuspended in 100 µL of HN buffer for further analysis and cryo-EM/cryo-ET sample preparation.

### Electron microscopy and cryogenic electron tomography

For negative staining TEM, an aliquot of 3 µL sample was applied to each carbon-coated copper grid (Electron Microscopy China, Beijing, China; http://www.emcn.com.cn). The samples were stained using 2% uranium acetate and imaged with a Tecnai 20 electron microscope operated at 200 kV (Thermo Fisher Scientific).

For cryo-EM and cryo-ET, a 3 µL aliquot of the virus sample was applied to a glow-discharged copper grid coated with holey carbon (R 2/1; Quantifoil, Germany). The samples were vitrified by plunge-freezing in a mixture of liquid ethane and propane at a 3:7 ratio using a homemade manual plunger. The frozen hydrated virions on the cryo-EM grids were imaged on a Titan Krios microscope (Thermo Fisher Scientific) operated at 300 kV. The microscope was equipped with an energy filter (slit width 20 eV; GIF Quantum LS, Gatan, CA) and a K3 direct electron detector (Gatan, CA). Tilt series of virions were recorded in super-resolution mode at a nominal magnification of 33,000× (corresponding to a calibrated pixel size of 2.605 Å at the specimen level). The tilt series were collected using a dose-symmetric scheme from –60° to 60° in 3° increments, with various defocus values ranging from –1.5 to –5.5 µm, using the acquisition software package serialEM (24). At each tilt angle, a movie consisting of six frames was recorded with an exposure time of 0.05 s per frame, resulting in a cumulative dose of 127.1 electrons/Å^2^ for the entire tilt series.

### Data processing

Frames in each movie were motion corrected using MotionCor2 (25) without dose weighting. The defocus value of the aligned images at each tilt angle was estimated using CTFFIND4 (26). Subsequent tilt-series alignment and reconstruction steps were performed in the framework of the IMOD version 4.11 software package (27) following steps: micrographs within each tilt series were aligned using patch tracking, and tomograms reconstructed via weighted back projection with a SIRT-like filter and 4× binning (10.42 Å per pixel).

For subtomogram averaging, the pre-fusion (triangular shape) and post-fusion (needle-like shape) conformations of the spike protein were identified based on their distinct differences in shape and width protruding from the virion envelope in the tomograms. We manually picked more than 200 pre-fusion and more than 50 post-fusion spike proteins using the two-point particle picking method in IMOD. Their coordinates were generated in tomoNet (28) and subsequently imported into RELION 4.0 (29) for subtomogram averaging. The particles were extracted as pseudo-subtomograms with a box size of 32 × 32 (4× binning, 10.42 Å per pixel). Subsequently, C3 symmetry was applied during 3D refinement. The resolutions for the final subtomogram averages were 37 Å and 38 Å for the pre- and post-fusion conformations, respectively. UCSF ChimeraX was used to visualize the averaged subtomogram maps. Atomic models (PDB accession codes 6XR8 and 6XRA [30]) of the pre- and post-fusion spike protein were fitted as rigid bodies to the corresponding densities using the Fit in Map tool (31).

### Statistical analysis

The data were analyzed with an unpaired Student *t*-test by Prism software (GraphPad). All of the data are shown as mean ± standard deviation (SD) or mean ± standard error of the mean (SEM) from three independent experiments; ∗*P* ≤ 0.05*,* ∗∗*P ≤* 0.01, ∗∗∗*P ≤* 0.001.

## RESULTS

### Hydrogen peroxide efficiently inactivates SARS-CoV-2

**Fig 1:**
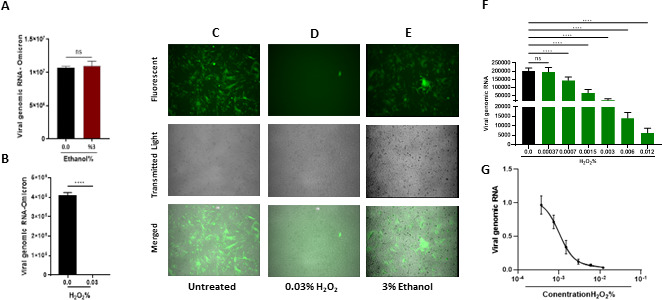
The efficacy of H_2_O_2_ in inhibition of infection with SARS-CoV-2. (**A and B**) Comparison of the ethanol and H_2_O_2_ potency in inhibition of SARS-CoV-2 infection. RT-qPCR analysis of RNA extracted from HeLa-ACE-2 cells infected with SARS-CoV-2 Omicron treated with (**A**) 3% ethanol or (**B**) 0.03% H_2_O_2_ to titrate the viral RNA. (**C and E**) Fluorescent microscopy images from HeLa-ACE-2 cells infected with SARS-CoV-2 expressing GFP which were (**C**) left untreated used as control, (**D**) treated with 0.03% H_2_O_2_, or (**E**) treated with 3% ethanol for 24 hours. (**F**) Quantification of RNA extracted from HeLa-ACE-2 cells infected with SARS-CoV-2 treated with serially diluted H_2_O_2_. Cells were harvested at 24 HPI, the viral RNA was extracted from virions released to the supernatant, and the viral RNA was titrated by RT-qPCR (**G**). Data from panel F were used to determine the IC_50_ of H_2_O_2_ in inhibiting the infection with SARS-CoV-2 in HeLa-ACE-2. All data are means ± SEM; ∗∗∗*P* < 0.001, ∗∗∗∗*P* < 0.0001.

To determine the efficacy of H_2_O_2_ in inactivation of SARS-CoV-2, viral particles of SARS-CoV-2 carrying the GFP gene (23) were exposed to either 3% (651 µM) ethanol or 0.03% (8.8 µM) H_2_O_2_. The treated particles were then used to infect the HeLa-ACE-2 cells for 24 hours. Quantification of viral RNA isolated from the infected cells by RT-qPCR indicated that 651 µM ethanol could not inhibit viral infection (Fig. 1A). By contrast, treatment of viral particles with 8.8 µM H_2_O_2_ significantly inhibited SARS-CoV-2 replication as shown in Fig. 1B. Furthermore, the fluorescent microscopy images also indicated that unlike H_2_O_2_ , treatment of SARS-CoV-2 virions with 651 µM ethanol, did not have any antiviral effect against SARS-CoV-2 (Fig. 1C through E). Next, we treated SARS-CoV-2 particles with serially diluted H_2_O_2_. These H_2_O_2_-treated viral particles were subsequently utilized as the virus inoculum to infect HeLa-ACE-2 cells for 1 hour. The infected cells were harvested at 24 HPI, and RNA samples were isolated from the cell lyase and subjected to RT-qPCR. The results indicated that a concentration as low as 0.003% of H_2_O_2_ could significantly suppress SARS-CoV-2 (Fig. 1F; Fig. S1A). The RT-qPCR-based titration of viral particles identified the 50% inhibitory concentration (IC_50_) of H_2_O_2_ in inhibiting the infection of SARS-CoV-2 in HeLa-ACE-2 cells to be as low as 0.0015% (Fig. 1G; Fig. S1B) which is far from its 50% cytotoxicity concentration (CC50) (Fig. S1C). These data indicate that H_2_O_2_ is a very potent antiviral agent against SARS-CoV-2, and its IC_50_ of 0.0015% is much lower than the 3% concentration commonly used for dental hygiene.

**Fig 2:**
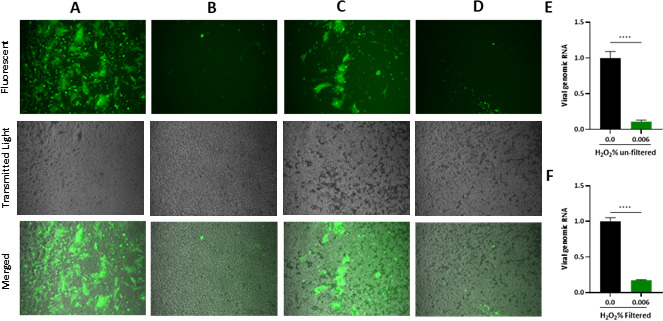
Dissecting the effect of H_2_O_2_ on the viability of cells and infectivity of SARS-CoV-2 particles. (**A–D**) Fluorescent microscopy images from HeLa-ACE-2 cells infected with SARS-CoV-2-expressing GFP for 24 hours which were (**A**) left untreated and unfiltered used as control, (**B**) treated with 0.006% H_2_O_2_ and unfiltered, (**C**) left untreated and filtered used as control, or (**D**) treated with 0.006% H_2_O_2_ and filtered. (**E**) Titration of viral RNA extracted from infected cells under panel A and B conditions. (**F**) Titration of viral RNA extracted from infected cells under panel C and D conditions. All data are means ± SEM; ∗∗∗*P* < 0.001, ∗∗∗∗*P* < 0.0001.

Next, we investigated whether the observed outcome of H_2_O_2_ inhibition of SARS-CoV-2 was solely due to the direct action of H_2_O_2_ on the virus or if it was also due to its effects on the host cells’ viability. To achieve this, SARS-CoV-2 virus particles, whether treated or untreated with H_2_O_2_, were passed through an ultra-centrifugal filter unit to clean out the viral particles from H_2_O_2_ before infecting cells. The filter was washed three times with the wash buffer, and the eluted viral particles were used to infect the HeLa-ACE-2 cells. At 24 HPI, microscopic images were taken of the infected cells under the indicated treatment. The results demonstrated that similar to unfiltered samples (Fig. 2A and B), H_2_O_2_ significantly inhibited the SARS-CoV-2 infection (Fig. 2C and D). To further quantify the effect of H_2_O_2_ treatment, we used RT-qPCR analysis to compare the level of viral genomic RNA isolated from cells infected with the H_2_O_2_-treated unfiltered SARS CoV-2 and those isolated from cells infected with the unfiltered SARS-COV-2 (Fig. 2E). Similarly, the viral titer of H_2_O_2_-treated-filtered SARS CoV-2 samples was compared to the filtered SARS-COV-2 (Fig. 2F). The results indicated that H_2_O_2_ treatment strongly inhibited SARS-CoV-2 replication in both unfiltered and filtered samples. These data suggested that H_2_O_2_ suppresses the SARS CoV-2 infection through its direct effect on viral particles.

**Fig 3:**
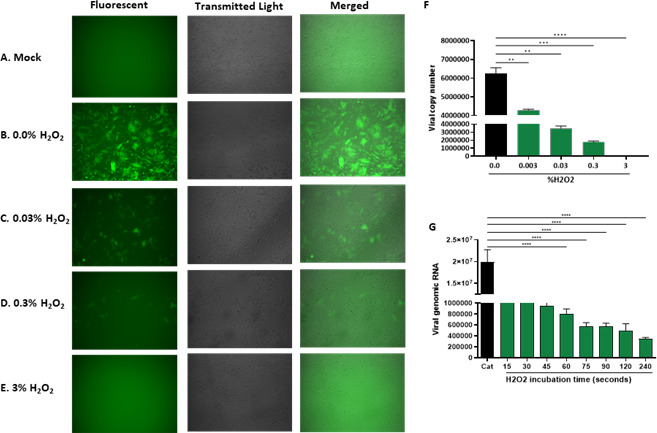
Hydrogen peroxide efficiently inactivates SARS-CoV-2. (**A–E**) Fluorescent microscopy images from HeLa-ACE-2 cells that were (**A**) left untreated-uninfected and used as a control or (**B–E**) infected with SARS-CoV-2 and treated with serially diluted H_2_O_2_ as indicated followed by catalase treatment. (**F**) The viral RNA was isolated from the viral particles released to the supernatant of the infected cells, and the copy number of viral RNA was quantified by RT-qPCR. (**G**) Determination of the minimum duration needed for hydrogen peroxide to effectively block SARS-CoV-2. HeLa-ACE-2 cells were infected with SARS-CoV-2 which were treated with 1.5% H_2_O_2_ at the indicated time followed by catalase treatment for 5 minutes. The viral RNA was isolated from the infected cells and quantified by RT-qPCR. All data are means ± SEM; ∗∗∗*P* < 0.001, ∗∗∗∗*P* < 0.0001.

To further exclude the cytotoxic effects of H_2_O_2_ on host cells and determine the kinetics for H_2_O_2_-mediated effects on viral particles, we took advantage of catalase, an enzyme that catalyzes the decomposition of hydrogen peroxide into water and oxygen. We first treated SARS-CoV-2 without or with different concentrations of H_2_O_2_ for 10 minutes and then inactivated H_2_O_2_ by adding catalase before infecting HeLa-ACE-2 cells. This step ensures that the effects observed after the catalase treatment are not due to residual hydrogen peroxide. As shown in Fig. 3A through E, pre-treatments of SARS-CoV-2 with H_2_O_2_ still effectively inhibited the SARS-CoV-2 replication in the HeLa-ACE-2 cells even though H_2_O_2_ had been inactivated by catalase before infecting cells. This antiviral effect of H_2_O_2_ is not due to its potential cytotoxicity as all HeLa-ACE-2 cells were viable after co-treatment with H_2_O_2_ and catalase as shown in Fig. S2, catalase mitigated the hydrogen peroxide-induced cellular damage and preserved the cell’s viability. To further corroborate these results, SARS-CoV-2 particles were serially diluted and double-treated with H_2_O_2_ followed by catalase treatment. HeLa-ACE-2 were infected with these treated SARS-CoV-2 particles for 24 hours. The viral RNA was isolated from the viral particles released to the supernatant of the infected cells, and the copy number of viral RNA was quantified (Fig.3F). The results revealed that even at a concentration as low as 0.3%, H_2_O_2_ serves as a highly effective barrier against SARS-CoV-2.

To identify the duration necessary for hydrogen peroxide to impede the SARS-CoV-2 replication, we treated SARS-CoV-2 particles with 1.5% H_2_O_2_ for the indicated times and then inactivated H_2_O_2_ with catalase for 5 minutes before infecting HeLa-ACE2 cells. Viral RNA was extracted from the virions and released to the supernatant of the infected cells at 24 HPI. The results of RT-qPCR indicated that 1.5% H_2_O_2_ inactivated more than 50% of the viral particles in 30 seconds (Fig. 3G). These data suggest that H_2_O_2_ can directly and rapidly inactivate SARS-CoV-2 viral particles.

### Hydrogen peroxide efficacy against infection with SARS-CoV-2 variants of concern

**Fig 4:**
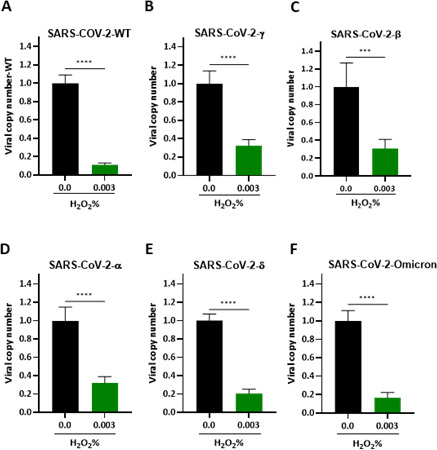
Hydrogen peroxide efficacy against infection with SARS-CoV-2 variants of concern. (**A–F**) HeLa-ACE-2 infection with SARS-CoV-2 variants exposed to H_2_O_2_. Viral RNA extracted from viral particles was released to the supernatant of the infected cells at 24 HPI and subjected to RT-qPCR. (**A**) Original SARS-CoV-2, (**B**) P.1 (Gamma), (**C**) B.1.351 (Beta), (**D**) B.1.1.7 (alpha), (**E**) B.1.617.2 (Delta), and (**F**) B.1.1.529 (Omicron). All data are means ± SEM; ∗∗∗*P* < 0.001, ∗∗∗∗*P* < 0.0001.

Being an RNA virus, SARS-CoV-2 constantly acquires mutations as it replicates. Mutations that are neutral or have only mild deleterious effects on the host may persist in the population, especially if they do not significantly impact the virus’s fitness. These mutations may accumulate over time, leading to genetic diversity within the virus population. These mutations may confer a fitness advantage to the virus, allowing it to replicate more efficiently, evade the host’s immune system, transmit more easily between individuals, or gain drug resistance. In such cases, these advantageous mutations may increase in frequency within the population over time, potentially leading to the emergence of new variants that are better adapted to their environment (4, 32, 33). To investigate the impact of H_2_O_2_ on the SARS-CoV-2 variants of concern, SARS-CoV-2 variants were exposed to 0.003% H_2_O_2_. The viral particles were then used to infect HeLa-ACE2 cells for 1 hour. The infected cells were collected at 24 HPI, and RNA extracted from the cell lysates was subjected to RT-qPCR analysis. The results showed that even at a concentration of 0.003%, H_2_O_2_ significantly suppressed the replication of SARS-CoV-2 variants of concern (Fig. 4A through F). Furthermore, to specifically investigate the impact of H_2_O_2_ on the viral particles rather than the cells, SARS-CoV-2 variants of concern were treated with H_2_O_2_ and then subjected to catalase treatment. The virus inoculum was introduced to HeLa-ACE-2 cells, and the infected cells were harvested at 24 HPI. The results obtained from this study revealed that all variants subjected to this study, including B.1.1.7 (alpha), B.1.351 (Beta), P.1 (Gamma), B.1.617.2 (Delta), and B.1.1.529 (Omicron), were remarkably inhibited by 0.03% of hydrogen peroxide (Fig. S3A through F). These data indicated that H_2_O_2_ effectively inhibited all tested SARS-CoV-2 variants of concern.

### PFA and H_2_O_2_ have different effects on the *in situ* structure of the spike protein

**Fig 5:**
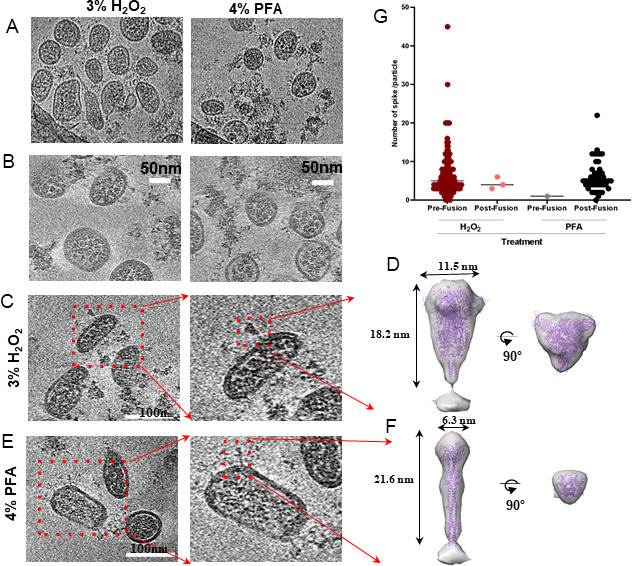
Cryo-EM and cryo-ET analyses of SARS-CoV-2 virions subjected to H_2_O_2_ or PFA treatments. (**A**) Representative cryo-EM images from SARS-CoV-2 particles treated with 3% H_2_O_2_ (left) or 4% PFA (right). (**B**) Representative density slices (thickness 5.21 nm) from cryo-ET tomograms of SARS-CoV-2 treated with 3% H_2_O_2_ (left) or 4% PFA (right). (**C–F**) Regions of cryo-ET reconstruction showing spike protein from SARS-CoV-2 particles treated with (**C and D**) 3% H_2_O_2_ or (**E and F**) 4% PFA. Subtomogram averaging (**D and F**) of spike proteins on the virions indicated the “RBD down” pre-fusion conformation (resolution, 37 Å) under (**D**) the 3% H_2_O_2_ treatment condition and (**F**) the “needle-like” post-fusion conformation (resolution, 38 Å) under the 4% PFA treatment. The subtomogram average maps are shown as semi-transparent surfaces in superposition with the purple ribbon diagrams of the (**D**) pre-fusion (PDB: 6XRA [30]) and (**F**) the post-fusion (PDB: 6XR8 [30]) atomic structures. (**G**) Statistics of different conformations of the spike protein induced by 3% H_2_O_2_ (more than 200 particles) or 4% PFA (more than 50 particles) treatments.

To investigate the structural state of the spike protein in SARS-CoV-2 particles inactivated by fixation, we infected Vero-E6 cells with SARS-CoV-2 and harvested the samples at 60 HPI. The harvested particles were then inactivated using either 4% PFA or 3% H_2_O_2_ and concentrated by ultracentrifugation. Subsequently, the viral particles were subjected to morphological examination using negative staining transmission electron microscopy (TEM) followed by cryo-EM images. Our analysis demonstrated that both negative TEM and cryo-EM images revealed variability in the sizes of viral particles under either 4% PFA or 3% H_2_O_2_ treatment (Fig. 5A; Fig. S4).

To visualize the 3D architecture of the virions and conformation of the spikes, we performed 3D reconstructions using cryo-ET (Fig. 5B). Our observations revealed that while virions exhibited various shapes, their overall shape and size were remarkably consistent under both 4% PFA and 3% H_2_O_2_ treatments as shown in the tomographic data (Fig. 5B). Notably, the viral membrane remained intact, exhibiting clear bilayer characteristics after fixation, which indicated that our treatments did not compromise the integrity of the viral membrane. Furthermore, the thickness of the bilayer was nearly identical between virions treated with 4% PFA and those treated with 3% H_2_O_2_ (Fig. 5C and E). Notably, the ribonucleoproteins (RNPs) were distinctly identifiable, and their distribution and characteristics were nearly identical between virions treated with 4% PFA and those treated with 3% H_2_O_2_ (Fig. 5C and E). This suggested that our treatments did not affect the RNPs based on our tomographic data. We also compared the spike protein under these two treatment conditions. Interestingly, in virions treated with 4% PFA, the spike protein exhibited a needle-like shape, measuring 21.6 nm in length and 6.3 nm in width, as seen in the tomogram slice (Fig. 5E). By contrast, those treated with 3% H_2_O_2_ displayed a triangular shape, measuring 18.2 nm in length and 11.5 nm in width (Fig. 5C). We hypothesized that these two distinct shapes of the spike proteins represented different conformations, suggesting that the fixation method may influence the spike protein conformation.

To quantify this observation, we analyzed the distribution of structural states of the spike protein on virions. Analysis of H_2_O_2_-inactivated virion particles revealed that the spike proteins predominantly adopted a pre-fusion conformation, characterized by the RBD in a down conformation, resembling a “trimer of hairpins.” By contrast, trimers on the viral particles inactivated with PFA displayed a post-fusion conformation (Fig. 5G) and Table S1.

To further determine the conformation of the spike proteins on the virion, we performed subtomogram averaging (STA). We manually selected 206 triangular-shaped and 54 needle-like particles from the data sets and processed them using RELION 4.0, resulting in the structures of the different shapes of the spike proteins with resolutions of 37 Å and 38 Å, respectively (Fig. 5D and F). While the low resolution limited the detailed structural insights, we observed significant differences in structural features between viral particles inactivated with 3% H_2_O_2_ and those inactivated with 4% PFA. We fitted pre- and post-fusion models into the maps (Fig. 5D and F). Fitting of S trimer atomic models into the cryo-ET density map confirmed that the triangular shape on the H_2_O_2_-treated viral particles corresponds to the pre-fusion spike protein (with the receptor-binding domain, the RBD, in a down conformation), while the needle-like shape on the PFA-treated viral particles represents the post-fusion spike protein. Specifically, S is in the “RBD down,” pre-fusion conformation under 3% H_2_O_2_ treatment, while on 4% PFA-treated SARS-CoV-2 particles, it closely resembled that of the post-fusion conformation of recombinant spike protein structures (30, 34).

## DISCUSSION

Hydrogen peroxide (H_2_O_2_) exhibits broad-spectrum antiviral activity, making it effective against a variety of viral pathogens, including enveloped and non-enveloped viruses (35) such as coronaviruses, influenza viruses, noroviruses, and other respiratory viruses (36, 37). Its rapid action and broad efficacy make it a valuable tool in preventing the transmission of viral infections in healthcare settings, public spaces, and households (38–41). The antiviral activity of hydrogen peroxide is attributed to its ability to generate reactive oxygen species (ROS), such as hydroxyl radicals and singlet oxygen, upon decomposition. However, the molecular mechanisms responsible for such broad antiviral effects of H_2_O_2_ are not understood. In the current study, we investigated the antiviral effects of H_2_O_2_ on SARS-CoV-2, established the effective dose and treatment time required for virus inactivation, and analyzed the impact of H_2_O_2_ treatment on distributions of S trimers *in situ* on the virion surface compared to PFA treatment under identical conditions, both in 2D with cryo-EM and in 3D with cryo-ET.

The SARS-CoV-2 spike protein (S) plays a crucial role in both cell entry and immune evasion. It is a major determinant of viral infectivity and pathogenesis (42). Notably, S undergoes extensive structural rearrangements during the entry process, transitioning from a pre-fusion to a post-fusion conformation. These conformational changes are crucial for the virus to establish infection. In its pre-fusion state, S exists as a trimeric glycoprotein protruding from the viral envelope. S in this state is primed for receptor engagement and subsequent fusion with the host cell membrane. In the post-fusion state, S adopts a more stable conformation characterized by a needle-like structure, with the fusion peptide inserted into the host cell membrane. The transition from the pre- to post-fusion conformation enables the fusion of viral and endosomal membranes, allowing the viral genomic RNA into the cytosol. Our cryo-EM, cryo-ET, and subtomogram average analyses showed that S on SARS-CoV-2 was in the post-fusion conformation when inactivated by PFA, but remained in the pre-fusion conformation with H_2_O_2_ treatment (Fig. 5). The results of this study indicate that H₂O₂ treatment of SARS-CoV-2 locks the spike (S) protein in the pre-fusion conformation, whereas PFA treatment predominantly stabilizes the S protein in the post-fusion state. These findings contrast with those of Yao et al. (18), Turoňová et al. (16), and Ke et al. (17), who reported that PFA treatment predominantly triggers the pre-fusion state of the S protein. Notably, SARS-CoV-2 infection of ACE2-overexpressing A549 cells followed by PFA fixation has been shown to promote a shift toward the post-fusion conformation (19). In addition, in Vero cells treated with PFA, both pre-fusion and post-fusion forms of the S protein have been observed (20).

It is well established that the methods used for viral sample preparation, inactivation, and fixation can influence the distribution of conformational states of the S protein, including the relative proportions of pre-fusion and post-fusion forms (15). The duration and conditions of PFA treatment, as determined by UCLA BSL3 regulations, were rigorous, involving 3 hours at room temperature, followed by 24 hours at 4°C, to ensure complete viral inactivation. By contrast, Yao et al. employed 48 hours at 4°C (18), while Ke et al. used 30 minutes at room temperature (15, 17). Furthermore, a very low MOI of 0.01 was used in our study to mimic more physiologically relevant conditions, where viral spread is typically slower and controlled. In addition, this strategy helps reduce the potential for defective viral particles (20).

Another consideration could be the viral strain used in each study. Different laboratories may work with distinct SARS-CoV-2 isolates, which could impact the results. For example, Song et al. showed that the Delta variant of SARS-CoV-2 has a significantly higher number of spike proteins on the virion compared to the wild-type (WT) strain. While both the delta variant and WT strain exhibit a higher ratio of pre-fusion to post-fusion spike proteins after PFA fixation, the Delta variant has a notably higher number of post-fusion spikes. In addition, E-beam inactivation significantly alters the ratio of post-fusion to pre-fusion spike proteins in both the Delta and WT strains (43). In the current study, a recombinant SARS-CoV-2 (icSARS-CoV-2-mNG) expressing mNeonGreen (23) was used.

H_2_O_2_ is known for its oxidative properties and likely has induced modifications, such as oxidation of cysteine residues within the RBD of S. Such oxidative modification may have locked the S trimer in its pre-fusion conformation, preventing the transition of S to the post-fusion state, an essential step for viral fusion and entry into host cells. The cysteine pairs (Cys336–Cys361, Cys379–Cys432, and Cys391–Cys525) in the core, and Cys480–Cys488 (Fig. S5) located at the distal end of the RBD, are known to play a pivotal role in stabilizing the spike structure (6). These cysteine residues may be modified by H_2_O_2_-mediated oxidation of S. Oxidation of the Cys480–Cys488 pair could potentially disrupt the conformation of the RBD, hindering ACE2 binding and favoring the pre-fusion state by preventing the conformational changes necessary for membrane fusion. In addition, Cys538–Cys590 near the S1/S2 cleavage site, Cys-617-Cys 649 in the S2 subunit, and Cys131–Cys166 in the N-terminal domain may also impact the S transition between pre-fusion and post-fusion states. The primary focus of the present study is to investigate the native spike protein in its bound state to the virion. We will continue to explore the role of H_2_O_2_ treatment on cysteine residues in RBD using the purified trimeric S protein in future studies.

Research on infectious SARS-CoV-2 virions must be performed in a BSL-3 facility. However, our BSL-3 facility is not equipped with a cryo-EM microscope. As a result, viral particles must be fully inactivated using an appropriate fixative before they can be safely transferred out of the BSL-3 lab for further analysis. Consequently, we are unable to compare the number of spike proteins on infectious virions to that on inactivated particles either with PFA or H₂O₂ treatment. The results in Table S1 show variability in spike counts in both PFA and H₂O₂-treated particles.

While H_2_O_2_ is primarily known as an oxidizing agent, its specific effects on protein structure and conformation may vary depending on other factors, including the local environment and the presence of other molecules or ions. Though not possible to evaluate due to the pleomorphic nature of their structures, interactions between H_2_O_2_ and other components of the SARS-CoV-2 particles or the surrounding environment may have also contributed to the observed anti-SARS-CoV-2 effect and the stabilization of the pre-fusion state of S. Such interactions include chemical reactions, structural modifications to lipids and viral genome, or/and changes in molecular interactions.

In conclusion, our study established the virucidal effects of H_2_O_2_ on SARS-CoV-2 and offers valuable insights into the structural basis of this effect. The broad-spectrum antiviral activity, rapid action, safety profile, and compatibility with various surfaces make hydrogen peroxide an asset in disinfection and sterilization efforts (44–46). While the virucidal activity of H_2_O_2_ against other coronaviruses has been investigated by others, the results presented here represent the first time when the impact of H_2_O_2_ on structural changes of SARS-CoV-2 spikes and the difference from that of PFA inactivation are systematically assessed.

## SUPPLEMENTAL MATERIAL

Fig. S1 mbio.03994-24-s0001.tiff Fluorescent microscopy and IC_50_ and CC_50_ of H_2_O_2_.

Fig. S2 mbio.03994-24-s0002.tiff IC_50_ of H_2_O_2_.

Fig. S3 mbio.03994-24-s0003.tiff Hydrogen peroxide efficacy against infection with SARS-CoV-2 variants of concern.

Fig. S4 mbio.03994-24-s0004.tiff Negative staining TEM images of virions treated with 3% H_2_O_2_ and 4% PFA.

Fig. S5 mbio.03994-24-s0005.tiff Key disulfide bonds that may facilitate the transition of the spike protein from its pre-fusion to post-fusion conformation.

Legends mbio.03994-24-s0006.docx Supplemental figure and table legends.

Table S1 mbio.03994-24-s0007.docx Spike count.

ASM does not own the copyrights to Supplemental Material that may be linked to, or accessed through, an article. The authors have granted ASM a non-exclusive, world-wide license to publish the Supplemental Material files. Please contact the corresponding author directly for reuse.
