# Supplementary figures and images for: Infectivity and structure of SARS-CoV-2 after hydrogen peroxide treatment
Source: mBio. 2025 Apr 21;16(5):e03994-24. doi: 10.1128/mbio.03994-24 (PMC12077155; doi:10.1128/mbio.03994-24)

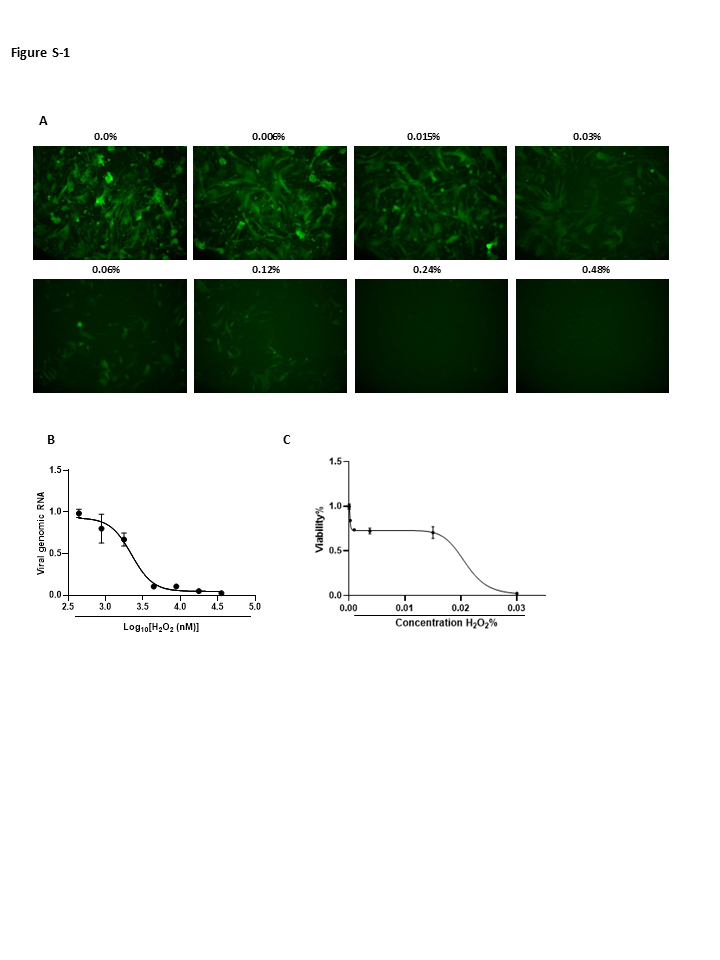

Supplement: Fig. S1 — Fluorescent microscopy and IC50 and CC50 of H2O2. [file mbio.03994-24-s0001.tiff]

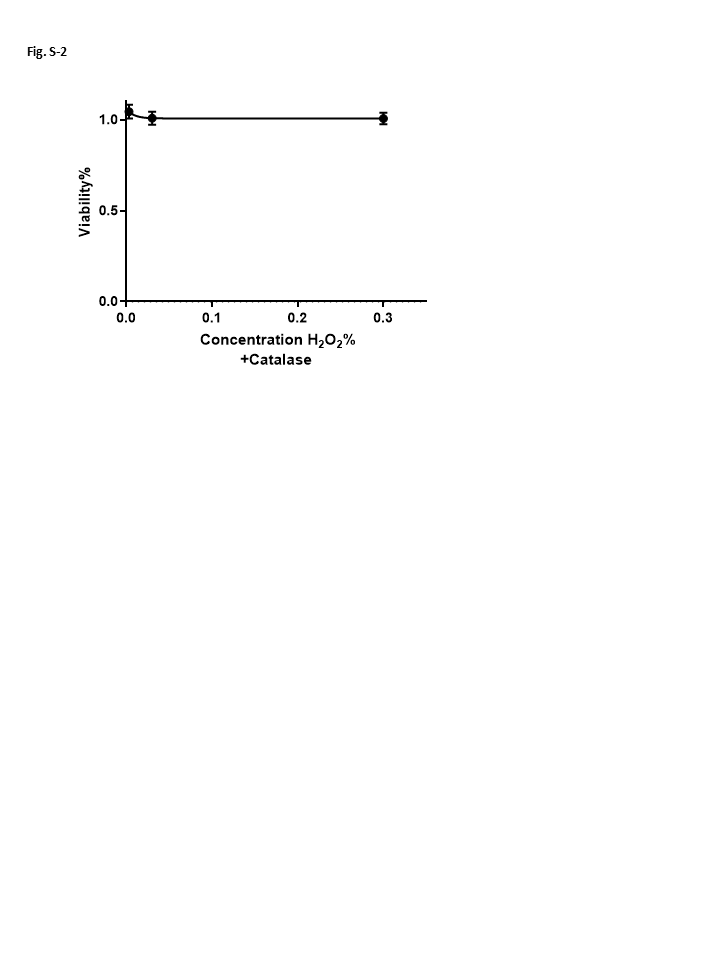

Supplement: Fig. S2 — IC50 of H2O2. [file mbio.03994-24-s0002.tiff]

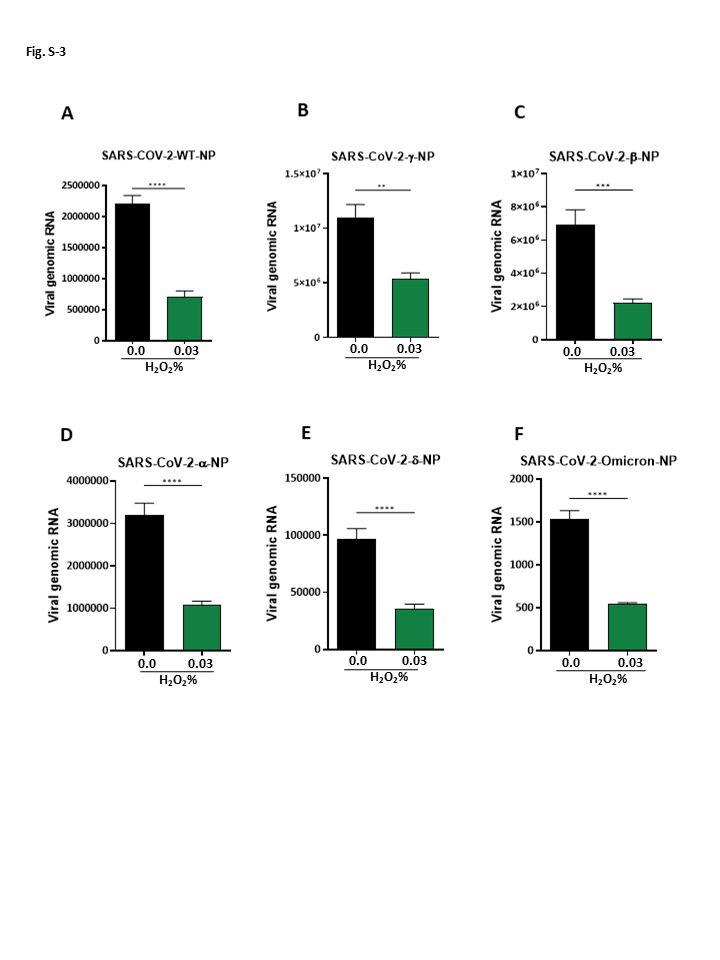

Supplement: Fig. S3 — Hydrogen peroxide efficacy against infection with SARS-CoV-2 variants of concern. [file mbio.03994-24-s0003.tiff]

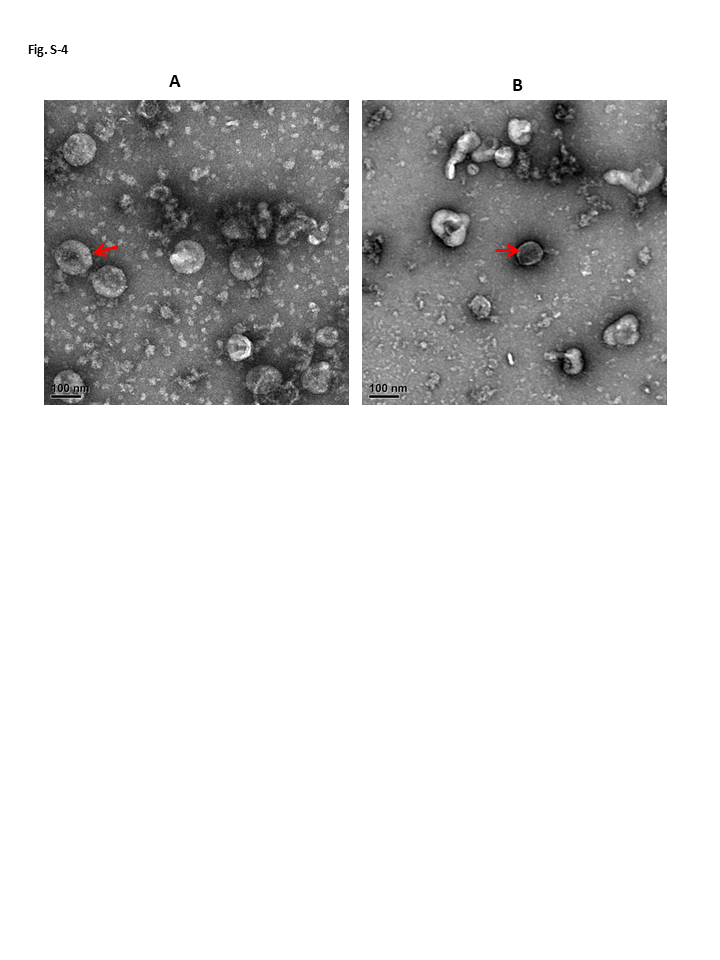

Supplement: Fig. S4 — Negative staining TEM images of virions treated with 3% H2O2 and 4% PFA. [file mbio.03994-24-s0004.tiff]

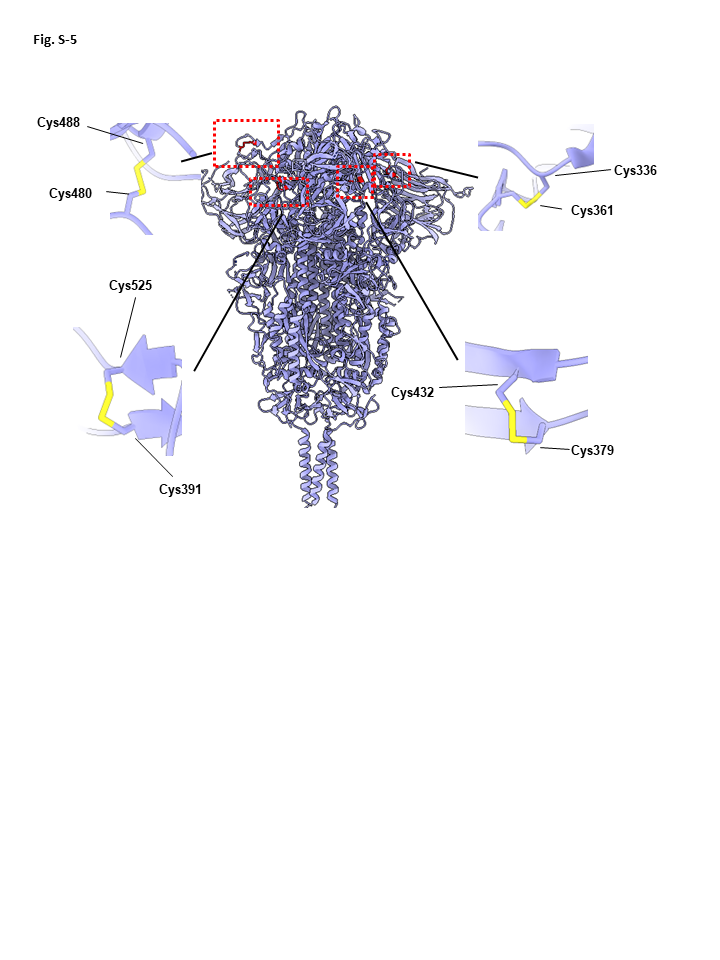

Supplement: Fig. S5 — Key disulfide bonds that may facilitate the transition of the spike protein from its pre-fusion to post-fusion conformation. [file mbio.03994-24-s0005.tiff]
