# Supplementary material for: Infectivity and structure of SARS-CoV-2 after hydrogen peroxide treatment
Source: mBio. 2025 Apr 21;16(5):e03994-24. doi: 10.1128/mbio.03994-24 (PMC12077155; doi:10.1128/mbio.03994-24)
Supplement: Legends — Supplemental figure and table legends. [file mbio.03994-24-s0006.docx]

**Supplementary Figure 1. (A)** Fluorescent microscopy images from Hela-ACE-2 cells infected with SARS-CoV-2 expressing GFP treated with serially diluted H_2_O_2_ as indicated, at 24 hours-post infection. (B) Determining the IC_50_ of hydrogen peroxide (H₂O₂) for inhibiting SARS-CoV-2 in Hela-ACE2 cells. (C) Identification of CC_50_ of H_2_O_2_ in Hela-ACE-2 cells.

**Supplementary Figure 2.**  Identification of the IC_50_ of H₂O₂ which was decomposed with catalase for inhibiting SARS-CoV-2 in Hela-ACE2 cells.

**Supplementary Figure 3**. Hydrogen peroxide efficacy against infection with SARS-CoV-2 variants of concern. Hela-ACE-2-infection with SARS-CoV-2 variants exposed to H₂O₂ followed by catalase treatment. Viral RNA extracted from viral particles released to the supernatant of the infected cells at 24HPI and subjected to RT-qPCR (A), original SARS-CoV-2 (B) P.1 (Gamma), (C) B.1.351 (Betta), (D) B.1.1.7 (alpha), (E) B.1.617.2 (Delta), and (F) B.1.1.529 (Omicron). All data are means ± SEM; ∗∗∗P < 0.001, ∗∗∗∗P < 0.0001.

**Supplementary Figure 4**. Negative staining TEM images of virions treated with 3% H₂O₂(left) and 4% PFA (right). Red arrows point to virions.

**Supplementary Figure 5.** The key disulfide bonds that may facilitate the transition of the spike protein from its pre-fusion to post-fusion conformation (PDB: 6XR8) are highlighted. These disulfide bonds are represented as red circles within the red-dotted box.

**Table 1.** Quantification of spike protein in the pre-fusion and post-fusion states in SARS-CoV-2 virions treated with 3% H₂O₂ or 4% PFA.
