# Supplementary material for: Infectivity and structure of SARS-CoV-2 after hydrogen peroxide treatment
Source: mBio. 2025 Apr 21;16(5):e03994-24. doi: 10.1128/mbio.03994-24 (PMC12077155; doi:10.1128/mbio.03994-24)
Supplement: Table S1 — Spike count. [file mbio.03994-24-s0007.docx]

| **Table 1**. Quantification of the number of spike protein in pre-or post-fusion state |
| --- |
| in the SARS-CoV-2 virions treated with 3% H₂O₂ or 4% PFA |

H₂O₂-treated SARS-CoV-2 particles

**Spike protein state**

| **Virion number** | **Prefusion spike number** | **Postfusion spike number** |
| --- | --- | --- |
| 1 | 4 |  |
| 2 | 1 |  |
| 3 | 1 |  |
| 4 | 3 |  |
| 5 | 4 |  |
| 6 | 3 |  |
| 7 | 4 |  |
| 8 | 5 |  |
| 9 | 4 |  |
| 10 | 4 |  |
| 11 | 4 |  |
| 12 | 4 |  |
| 13 | 5 |  |
| 14 | 1 | 4 |
| 15 | 6 |  |
| 16 | 6 |  |
| 17 | 3 |  |
| 18 | 2 |  |
| 19 | 4 |  |
| 20 | 9 |  |
| 21 | 6 | 2 |
| 22 | 3 |  |
| 23 | 9 |  |
| 24 | 3 |  |
| 25 | 6 |  |
| 26 | 5 |  |
| 27 | 1 |  |
| 28 | 5 |  |
| 29 | 3 |  |
| 30 | 6 |  |
| 31 | 3 |  |
| 32 | 1 |  |
| 33 | 5 |  |
| 34 | 1 |  |
| 35 | 3 |  |
| 36 | 3 |  |
| 37 | 5 |  |
| 38 | 3 |  |
| 39 | 3 |  |
| 40 | 4 |  |

| 41 | 3 |  |
| --- | --- | --- |
| 42 | 2 |  |
| 43 | 7 |  |
| 44 | 4 |  |
| 45 | 3 |  |
| 46 | 5 |  |
| 47 | 4 |  |
| 48 | 1 |  |
| 49 | 5 |  |
| 50 | 4 |  |
| 51 | 2 |  |
| 52 | 2 |  |
| 53 | 4 |  |
| 54 | 4 |  |
| 55 | 1 |  |
| 56 | 1 |  |
| 57 | 5 |  |
| 58 | 3 |  |
| 59 | 8 |  |
| 60 | 5 |  |
| 61 | 2 |  |
| 62 | 2 |  |
| 63 | 2 |  |
| 64 | 6 |  |
| 65 | 6 |  |
| 66 | 8 |  |
| 67 | 5 |  |
| 68 | 6 |  |
| 69 | 11 |  |
| 70 | 4 |  |
| 71 | 10 |  |
| 72 | 3 |  |
| 73 | 4 |  |
| 74 | 10 |  |
| 75 | 11 |  |
| 76 | 4 |  |
| 77 | 6 |  |
| 78 | 0 | 3 |
| 79 | 3 |  |
| 80 | 3 |  |
| 81 | 9 |  |
| 82 | 3 |  |
| 83 | 3 |  |
| 84 | 2 |  |
| 85 | 2 |  |
| 86 | 8 |  |
| 87 | 4 |  |
| 88 | 4 |  |
| 89 | 2 |  |

| 90 | 4 |  |
| --- | --- | --- |
| 91 | 2 |  |
| 92 | 5 |  |
| 93 | 5 |  |
| 94 | 5 |  |
| 95 | 3 |  |
| 96 | 5 |  |
| 97 | 3 |  |
| 98 | 6 |  |
| 99 | 3 |  |
| 100 | 9 |  |
| 101 | 45 |  |
| 102 | 8 |  |
| 103 | 10 |  |
| 104 | 5 |  |
| 105 | 30 |  |
| 106 | 8 |  |
| 107 | 2 |  |
| 108 | 5 |  |
| 109 | 6 |  |
| 110 | 4 |  |
| 111 | 1 |  |
| 112 | 4 |  |
| 113 | 2 |  |
| 114 | 6 |  |
| 115 | 2 |  |
| 116 | 3 |  |
| 117 | 3 |  |
| 118 | 7 |  |
| 119 | 7 |  |
| 120 | 5 |  |
| 121 | 14 |  |
| 122 | 12 |  |
| 123 | 6 |  |
| 124 | 6 |  |
| 125 | 4 |  |
| 126 | 1 |  |
| 127 | 2 |  |
| 128 | 12 |  |
| 129 | 5 |  |
| 130 | 10 |  |
| 131 | 7 |  |
| 132 | 7 |  |
| 133 | 10 |  |
| 134 | 4 |  |
| 135 | 8 |  |
| 136 | 7 |  |
| 137 | 11 |  |
| 138 | 2 |  |

| 139 | 11 |  |
| --- | --- | --- |
| 140 | 10 |  |
| 141 | 5 |  |
| 142 | 13 |  |
| 143 | 10 |  |
| 144 | 5 |  |
| 145 | 5 |  |
| 146 | 10 |  |
| 147 | 7 |  |
| 148 | 5 |  |
| 149 | 15 |  |
| 150 | 7 |  |
| 151 | 5 |  |
| 152 | 5 |  |
| 153 | 14 |  |
| 154 | 6 |  |
| 155 | 6 |  |
| 156 | 6 |  |
| 157 | 12 |  |
| 158 | 2 |  |
| 159 | 10 |  |
| 160 | 5 |  |
| 161 | 2 |  |
| 162 | 2 |  |
| 163 | 2 |  |
| 164 | 2 |  |
| 165 | 20 |  |
| 166 | 16 |  |
| 167 | 14 |  |
| 168 | 20 |  |
| 169 | 5 |  |
| 170 | 6 |  |
| 171 | 4 |  |
| 172 | 8 |  |
| 173 | 6 |  |
| 174 | 2 |  |
| 175 | 4 |  |
| 176 | 13 |  |
| 177 | 1 |  |
| 178 | 2 |  |
| 179 | 5 |  |
| 180 | 15 |  |
| 181 | 4 |  |
| 182 | 1 |  |
| 183 | 3 |  |
| 184 | 5 |  |
| 185 | 4 |  |
| 186 | 2 |  |
| 187 | 3 |  |

| 188 | 4 |  |
| --- | --- | --- |
| 189 | 3 |  |
| 190 | 4 |  |
| 191 | 1 |  |
| 192 | 4 |  |
| 193 | 3 |  |
| 194 | 10 |  |
| 195 | 6 |  |
| 196 | 20 |  |
| 197 | 5 |  |
| 198 | 4 |  |
| 199 | 6 |  |
| 200 | 4 |  |
| 201 | 5 |  |
| 202 | 4 |  |
| 203 | 6 |  |
| 204 | 2 |  |
| 205 | 6 |  |

PFA-treated SARS-CoV-2 particles

**Spike protein state**

| **Virion number** | **Pre-fusion spike number** | **Postfusion spike number** |
| --- | --- | --- |
| 1 |  | 5 |
| 2 |  | 8 |
| 3 |  | 4 |
| 4 |  | 12 |
| 5 |  | 12 |
| 6 |  | 10 |
| 7 |  | 3 |
| 8 |  | 12 |
| 9 |  | 4 |
| 10 |  | 5 |
| 11 |  | 6 |
| 12 |  | 5 |
| 13 |  | 5 |
| 14 |  | 5 |
| 15 |  | 5 |
| 16 |  | 4 |
| 17 |  | 5 |
| 18 |  | 7 |
| 19 |  | 12 |
| 20 |  | 6 |
| 21 |  | 2 |
| 22 | 1 | 0 |
| 23 |  | 7 |
| 24 |  | 4 |
| 25 |  | 4 |
| 26 |  | 6 |
| 27 |  | 2 |
| 28 |  | 2 |
| 29 |  | 4 |
| 30 |  | 2 |
| 31 |  | 8 |
| 32 |  | 5 |
| 33 |  | 5 |
| 34 |  | 2 |
| 35 |  | 5 |
| 36 |  | 5 |
| 37 |  | 12 |
| 38 |  | 10 |
| 39 |  | 6 |
| 40 |  | 1 |

| 41 |  | 13 |
| --- | --- | --- |
| 42 |  | 4 |
| 43 |  | 6 |
| 44 |  | 7 |
| 45 |  | 22 |
| 46 |  | 6 |
| 47 |  | 5 |
| 48 |  | 4 |
| 49 |  | 3 |
